# Cortical Tremor (CT) with coincident orthostatic movements

**DOI:** 10.1186/s40734-014-0013-0

**Published:** 2015-02-02

**Authors:** Pichet Termsarasab, Steven J Frucht

**Affiliations:** Department of Neurology, Movement Disorder Division, 5 East 98th St, New York, NY 10029 USA; Icahn School of Medicine at Mount Sinai, New York, NY USA

**Keywords:** Cortical tremor, Orthostatic tremor, Levetiracetam

## Abstract

Cortical tremor (CT) is a form of cortical reflex myoclonus that can mimic essential tremor (ET). Clinical features that are helpful in distinguishing CT from ET are the irregular and jerky appearance of the movements. We report two patients with CT with coexisting orthostatic movements, either orthostatic tremor (OT) or myoclonus, who experienced functional improvement in both cortical myoclonus and orthostatic movements when treated with levetiracetam.

**Electronic supplementary material:**

The online version of this article (doi:10.1186/s40734-014-0013-0) contains supplementary material, which is available to authorized users.

## Background

Cortical tremor (CT) is a syndrome described in the literature by multiple names, including familial cortical tremor, benign adult familial myoclonic epilepsy, familial adult myoclonic epilepsy, autosomal dominant cortical myoclonus and epilepsy, and familial cortical myoclonic tremor among others [1]. We will use the term CT in this paper for consistency. Clinically small-amplitude, irregular, jerky movements affect the distal limbs in posture and action. The disorder can mimic essential tremor (ET), and many patients are incorrectly diagnosed with ET [2]. Electrophysiologic studies however reveal features of cortical reflex myoclonus including cortical spikes prior to myoclonus on back-averaging, giant somatosensory-evoked potentials (SEPs), and enhanced C-reflex [3].

Although tremulous movements in the legs have been described in some case reports of CT [4,5], to our knowledge coincident orthostatic movements, have not been reported in these patients. We present two patients with CT with coexisting orthostatic movements, either orthostatic tremor (OT) or myoclonus, both of whom enjoyed a favorable response to treatment of CT and orthostatic movements with levetiracetam.

## Case presentation

### Patient 1

**Figure 1:**
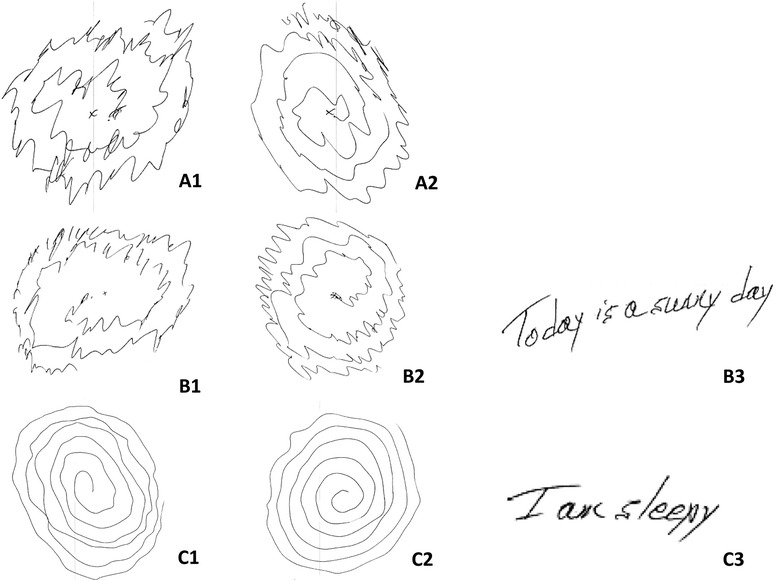
**Panel A, B and C demonstrate spirals and handwriting of patient 1 in January 2013, February 2013, and May 2013, respectively.** She was on no medication in January 2013, levetiracetam 2,000 mg/day in February 2013, and levetiracetam 2,000 mg/day and primidone 100 mg/day in May 2013. Panels A1, B1 and C1 are spirals from the left hand, and panels A2, B2 and C2, are from the right. Panels B3 and C3 are handwriting from the right hand. There is improvement of her hand spirals and handwriting over time.

A 61-year-old woman was referred for evaluation of tremulous movements of her hands that had progressively worsened over the past five years. Movements could not be voluntarily suppressed and were severe enough to cause functional disability (Additional file 1: Video 1). In addition, she had instability when standing, and noticed that this feeling of instability instantly disappeared when she began to walk or if she held onto a wall. There was no history of epilepsy. Examination revealed frequent and moderate amplitude myoclonic jerks affecting both hands at rest. Myoclonus was worse with posture and action, such as when performing finger-to-nose testing. There was a superimposed, regular, oscillatory action tremor of both hands with approximately 6-Hz ET-like frequency (Additional file 1: Video 1). This tremor was also visible on Archimedes spiral (Figure 1A). The tremor occurred in the same pattern without inconsistency or incongruency. When she stood, there was a very low amplitude high frequency tremor of her legs that was visible and palpable. Leg tremor abated immediately when she put her hand on the wall, and disappeared when she walked. There were no signs of parkinsonism. MRI of the brain and routine EEG were unremarkable.

A diagnosis of CT and coincident orthostatic movements (likely OT) was ascertained clinically. Physiologic confirmation was unfortunately not available. She was started on levetiracetam with a slow titration to 2,000 mg/day, with marked improvement of both her myoclonus, the ET-like movements of her hands (Figure 1B and C), and symptoms of OT (Additional file 2: Video 2). Primidone was added and titrated up slowly to 100 mg/day with more improvement in tremulous movements of her hands. However, she could not tolerate it due to sedation, and the dose was decreased to 50 mg/day. Clonazepam was ineffective.

### Patient 2

An 85-year-old woman presented with symptoms of unsteadiness when standing, leading to multiple falls. She had also noticed that on several occasions her left hand flung out uncontrollably. On examination, low amplitude myoclonic jerks of the left arm occurred with posture and action. Twenty seconds after standing up, she developed irregular jerky movements of the left leg which made her very unsteady. These movements disappeared when she reached out for the wall or when she began to walk, but recurred 20 seconds after she stopped walking (Additional file 3: Video 3). There were no signs of parkinsonism. A DAT scan obtained prior to evaluation by the referring neurologist was normal.

A clinical diagnosis of CT with likely coincident OT was made; physiologic confirmation was not available. She was treated with levetiracetam with a slow titration up to 1,500 mg/day. Significant improvement in both cortical tremor and OT was noted, but she developed depression. The dose was decreased to 1,000 mg/day with resolution of depression, and symptoms of CT and OT remained well controlled (Additional file 4: Video 4).

## Discussion

We present two patients whose histories and examinations support the diagnosis of CT. Both patients also had symptoms and signs of orthostatic movements, and both conditions responded to treatment with levetiracetam. OT is phenomenologically distinct from cortical myoclonus. Orthostatic myoclonus has been reported as a differential diagnosis of OT [6,7]; we recognize that electrophysiology would be required to distinguish these two. The pathophysiology of orthostatic myoclonus is still not well understood, and the link between cortical reflex myoclonus and orthostasis myoclonus remains unknown. In addition, the differential diagnosis in our second patient includes late-onset asymmetric myoclonus in which cortical myoclonus is an electrophysiologic hallmark [8]. However, due to lack of electrophysiology, we cannot confirm if the left leg movements were indeed compatible with orthostatic tremor or myoclonus. Late-onset asymmetric myoclonus is very rare, and the association between CT and this entity is unknown.

Although postural hand tremor can be seen in OT, it is usually mild and present only when standing, unlike the myoclonic movements of the arms in our patients. The tremor of OT emerges after a latency with standing and markedly improves or immediately disappears with walking or touching a wall. OT is characterized by a 13–18 Hz tremor of both legs that may not be visible, and is considered to be one of the highest frequency tremors [9].

Characteristic clinical clues for the diagnosis of CT are tremulous small amplitude movements in the distal limbs that are jerky and arrhythmic. Although we did not have physiologic confirmation, the appearance of the movements and their dramatic response to levetiracetam supports the likelihood that they were indeed myoclonic. The distal predominance and triggering with action and intention are other clues to a likely cortical origin of the myoclonic jerks. Electrophysiologic testing would have been helpful in identifying characteristic features such as cortical spikes prior to myoclonus on back-averaging technique, giant SEPs, and enhanced C-reflex. Neither patient had a family history of a similarly affected relative, and neither one had a history of seizures, commonly seen in this condition [1].

Levetiracetam is an antiepileptic medication that binds to synaptic vesicle glycoprotein 2A (SV2A) in the brain, which in turn inhibits presynaptic calcium channel and neuronal activities. Levetiracetam possesses efficacy in treating cortical myoclonus, and the response to levetiracetam in our patients supports the diagnosis of CT. Although some ET patients may experience improvement with levetiracetam, the degree of improvement in our patients was quite striking, and indeed two randomized studies failed to show improvement of tremor with the drug [10,11].

Interestingly, patient 1 also has improvement in tremulous movements of her hands with primidone. This does not necessarily reflect the diagnosis of essential tremor. Primidone acts in the central nervous system, possibly affecting voltage-gated sodium channels, although the exact mechanism of action is unknown. It was originally used as an epileptic agent. Thus, we are not surprised that her cortical tremor was improved by primidone as well.

The coincident improvement of OT in our patients deserves comment. The pathophysiology of OT is incompletely understood. Abnormalities in central cerebello-thalamo-cortical oscillatory networks have been proposed [12]. The reduction of calcium currents by levetiracetam [13] may indirectly enhance GABA in this network, which may lead to reduction of OT. Although levetiracetam was found to be ineffective in one double-blind crossover study in OT [14], anecdotally some OT patients respond to levetiracetam therapy, as did both of our patients.

Establishing a correct diagnosis of both CT and OT is important for optimal treatment. Misinterpretation of tremulous movements as ET may lead to treatment with β-blockers which are typically ineffective in CT and OT [15]. Medications that are reported to be effective in OT include clonazepam and gabapentin. For CT, in addition to levetiracetam, clonazepam and valproic acid may also be effective.

## Conclusion

We report the two cases of CT with co-existing orthostatic movements, possibly OT. Levetiracetam may be an effective treatment options in these patients. Further study in larger number of patients with CT, and electrophysiology studies may be utilized to confirm the diagnosis of OT, or increase sensivity in detecting OT in CT patients with unsteadiness.

## Consent

Written informed consent was obtained from the patients for publication of this case report and any accompanying images. A copy of the written consent is available for review by the Editor-in-Chief of this journal.

## Additional files

Additional file 1: Video 1. Patient 1 had mild-to-moderate kinetic and postural greater than rest “tremor” of both hands. Her “tremor” was irregular and jerky, and in fact, it was cortical myoclonus. There was no stimulus sensitivity to somesthetic stimuli when examining with a pin. She felt imbalance when standing, and this feeling abated when walking. When she stopped walking and stood still, she felt unsteady again which was better when she touched the wall with her hand. When she wrote or drew a spiral, there was myoclonus visible in both hands. Additional file 2: Video 2. Video demonstrates her examination when patient 1 was on levetiracetam 2,000 mg/day. There was improvement of myoclonus in both hands, at rest and with action. In addition, there was improvement of orthostatic tremor and she felt more steady when standing. Myoclonus in both hands when writing or drawing a spiral was also improved. Additional file 3: Video 3. Patient 2 demonstrated mild postural and terminal kinetic “tremor” in both hands. The “tremor” was irregular and jerky, not typical for essential tremor. The phenomenology is indeed myoclonus. She felt unsteady when standing, and better when walking, touching the wall or holding the examiner’s hand or finger. Additional file 4: Video 4. Video demonstrates the examination when patient 2 was on levetiracetam 1,000 mg/day. There was improvement in myoclonus of her hands, and orthostatic tremor. She felt more steady when walking.
